# Estimated sdLDL-C for predicting high-risk coronary plaque features in psoriasis: a prospective observational study

**DOI:** 10.1186/s12944-023-01819-x

**Published:** 2023-04-27

**Authors:** Alexander V. Sorokin, Nidhi Patel, Haiou Li, Christin G. Hong, Maureen Sampson, Ross O’Hagan, Elizabeth M. Florida, Heather L. Teague, Martin P. Playford, Marcus Y. Chen, Nehal N. Mehta, Alan T. Remaley

**Affiliations:** 1 Section of Inflammation and Cardiometabolic Diseases, Cardiovascular Branch, National Heart, Lung and Blood Institute, National Institutes of Health, 9000 Rockville Pike, Bldg 10, Clinical Research Center, Room 5-5150, Bethesda, MD 20892 USA; 2 Section of Lipoprotein Metabolism, Translational Vascular Medicine Branch, Lung and Blood Institute, National Heart, National Institutes of Health, Bethesda, MD USA

**Keywords:** Lipids, Atherosclerosis, Inflammation, Small dense LDL-C, Psoriasis, Coronary plaque

## Abstract

**Background:**

Psoriasis (PSO) is a skin disorder with systemic inflammation and high coronary artery disease risk. A distinct lipid phenotype occurs in psoriasis, which is characterized by high plasma triglycerides (TGs) with typically normal or even low LDL-C. The extent to which cholesterol on LDL subfractions, such as small dense LDL-C (sdLDL-C), are associated with vulnerable coronary plaque characteristics in PSO remains elusive.

**Methods:**

A recently developed equation for estimating sdLDL-C from the standard lipid panel was utilized in a PSO cohort (n = 200) with 4-year follow-up of 75 subjects. Coronary plaque burden was assessed by quantitative coronary computed tomography angiography (CCTA). Multivariate regression analyses were used for establishing associations and prognostic value of estimated sdLDL-C.

**Results:**

Estimated sdLDL-C was positively associated with non-calcified burden (NCB) and fibro-fatty burden (FFB), which remained significant after multivariate adjustment for NCB (β = 0.37; *P* = 0.050) and LDL-C adjustment for FFB (β = 0.29; *P* < 0.0001). Of note, total LDL-C calculated by the Friedewald equation was not able to capture these associations in the study cohort. Moreover, in the regression modelling estimated sdLDL-C was significantly predicting necrotic burden progression over 4 years follow-up (*P* = 0.015), whereas LDL-C did not. Finally, small LDL particles (S-LDLP) and small HDL particles (S-HDLP), along with large and medium TG-rich lipoproteins (TRLPs) had the most significant positive correlation with estimated sdLDL-C.

**Conclusions:**

Estimated sdLDL-C has a stronger association than LDL-C with high-risk features of coronary atherosclerotic plaques in psoriasis patients.

**Clinical trial registration:**

URL: https://www.clinicaltrials.gov. Unique identifiers: NCT01778569

**Supplementary Information:**

The online version contains supplementary material available at 10.1186/s12944-023-01819-x.

## Introduction

Chronic inflammatory disorders, such as psoriasis (PSO), are now considered as risk-enhancing conditions in patients with intermediate atherosclerotic cardiovascular disease (ASCVD) risk, leading to a recommendation of the possible use of lipid-lowering therapy in this cohort of patients [1]. According to a recent populational study, more than 7.5 million adults have psoriasis in the United States [2] often associated with ASCVD [3] and metabolic dysfunction [4]. One of the reasons underlying this association resides in lipoprotein metabolism dysregulation caused by persistent inflammation and impaired immune response in chronic inflammatory conditions like psoriasis [5]. Interestingly, these patients often have a significant decrease in total cholesterol and in cholesterol on Low-Density Lipoproteins (LDL-C) in the 5 years preceding psoriasis onset [6], whereas at the time of disease progression, plasma triglycerides (TGs) also become elevated [7]. Abnormalities in triglyceride metabolism have been attributed to higher prevalence of metabolic syndrome in psoriatic population, which is characterized by lower LDL-C levels, impaired glucose tolerance and associated obesity [4]. Thus, given the higher incidence of ASCVD in psoriasis patients, further investigation of psoriasis-specific lipid phenotype and how it relates to ASCVD risk may be valuable for identifying new biomarkers in this population.

Pharmacological reduction of LDL-C is the main tool in the primary prevention for ASCVD; however, other lipid targets have been recently investigated to address the issue of residual atherosclerotic risk in patients [8]. Starting in the early 1990s, the differential association of LDL subfractions, such as large buoyant LDL (lbLDL) and small dense LDL (sdLDL) with CVD risk, as measured by tedious research methods like ultracentrifugation and electrophoresis, were first reported [9]. Subsequently, nuclear magnetic resonance (NMR) spectroscopy was developed for measuring LDL particle number (LDL-P) and LDL subfractions, and it has been extensively investigated in large populational studies, such as the Multi-Ethnic Study of Atherosclerosis (MESA) [10]. More recently, the possible clinical utility of a direct assay for the cholesterol content of sdLDL (sdLDL-C) for CVD risk was evaluated using fully automated homogeneous assays on standard clinical chemistry analyzers [11]. These studies have shown that sdLDL-C is frequently elevated in patients with chronic inflammatory conditions even when LDL-C is not elevated, including psoriasis [12], coronary artery disease (CAD) [13], diabetes [14] and metabolic syndrome [15].

Although association studies do not necessarily indicate that sdLDL-C is causally related to atherosclerosis, sdLDL does have several biochemical and biophysical properties that could increase its ability for causing atherosclerosis. For example, sdLDL has a higher susceptibility to oxidation in vitro [16] and more promptly enters the arterial wall because of its smaller size, where it also firmly binds to proteoglycans [17] than lbLDL. Indeed, a possible major contribution of small LDL particles to the observed CAD in psoriasis patients has been previously shown [18]. Even if sdLDL-C is not mechanistically linked to CVD, it is known to positively associate with markers of chronic inflammation (hs-CRP) [19] and thrombosis, (D-dimer) [20], both of which contribute to the pathogenesis of plaque formation. Finally, sdLDL levels are also tightly connected to triglyceride-rich lipoproteins (TRLPs) metabolism and TRLP-cholesterol (TRLP-C), which is sometimes defined as remnant-cholesterol. Both sdLDL-C and TRLP-C were reported to be better predictors of CVD risk than LDL-C [21] and both are associated with increased triglycerides and low High-Density Lipoprotein-Cholesterol (HDL-C) [22].

Based on the aforementioned clinical and mechanistical observations, we hypothesized that sdLDL-C may also be a useful biomarker for CAD in psoriasis patients. Recently, a new equation for estimating sdLDL-C has been successfully applied for improving CVD risk stratification in a general population study [23]; however, its clinical utility in psoriasis has never been examined. The equation only depends on the same parameters measured in the standard lipid panel for estimating LDL-C by the Friedewald and other LDL-C equations. Hence, it would be relatively easy to use sdLDL-C for risk stratification, because it does not involve any additional lab testing and only utilizes tests that already routinely used for ASCVD risk assessment.

In this study, we, therefore, examined whether estimated sdLDL-C has any advantage over estimated LDL-C, the main univariate metric currently used to manage the use of lipid-lowering therapy in psoriasis patients. We found a stronger association of estimated sdLDL-C than estimated LDL-C with high-risk features of coronary atherosclerotic plaques as determined by quantitative coronary computed tomography angiography (CCTA) in patients with psoriasis, supporting future research on the possible clinical utility of sdLDL-C as an ASCVD biomarker.

## Materials and methods

### Study design and overview

#### Psoriasis cohort

A total of 341 consecutive patients with psoriasis were examined from the ongoing longitudinal cohort study recruited between February 2013–2020 (PACI, The Psoriasis, Atherosclerosis, and Cardiometabolic Disease Initiative) (Supplemental Fig. 1). Participants were > 18 years of age and underwent blood draw and CCTA imaging at baseline, 1-year and 4-year follow‐up. Participants with psoriasis were required to have a formal diagnosis of plaque psoriasis by a dermatologist and had severity of skin disease assessed based on the Psoriasis Area Severity Index (PASI) score. Participants were excluded if they were pregnant or had severe renal disease (eGFR < 30 mL/min/1.73m^2^). A complete list of inclusion/exclusion criteria is listed at ClinicalTrials.gov, NCT01778569.

The data that support the findings of this study are available from the corresponding author upon request. Strengthening the reporting of observational studies in epidemiology (STROBE) guidelines were followed for reporting the findings [24].

### Clinical and biochemical measurements

Psoriasis-specific treatment at baseline was patient-reported and defined as use of any of the following therapeutics in the 3 months prior to the baseline visit: systemic therapy (steroids or methotrexate), biologic therapy (adalimumab, etanercept, ixekizumab, secukinumab and ustekinumab), statins, light therapy, and topical treatments were recorded.

Peripheral blood from the enrolled subjects was collected after overnight fasting in EDTA coated tubes and centrifuged for 20 min at 3500 rpm, 4 °C. Obtained plasma was aliquoted and immediately stored at – 80^o^C until further analysis without being exposed to a freeze-thaw cycle. Traditional plasma lipid parameters consisted of total cholesterol (TC), HDL-C and triglycerides (TG) levels, which were measured enzymatically on the Cobas 6000 analyzer (Roche Diagnostics, IN, USA). Unless otherwise indicated, LDL-C refers to cholesterol on LDL as calculated by the Friedewald equation. Remnant cholesterol was estimated as fasting TC minus LDL-C minus HDL-C. ApoA-I and ApoB concentrations were measured by automated turbidometric immunoassays on the Cobas 6000 analyzer (Roche Diagnostics, IN, USA). Other plasma biochemical measurements, including hsCRP, were also performed on a Cobas 6000 analyzer (Roche Diagnostics, IN, USA) in the NIH Clinical Center (Bethesda, MD).

Results of the standard lipid panel were used to generate formula-based estimates of large buoyant LDL cholesterol (lbLDL-C) and small dense LDL cholesterol (sdLDL-C) as reported previously [23], by utilizing an equation and software freely available at the following website: https://figshare.com/articles/software/Sampson_sdLDLC_Equation_Calculator_xlsx/12888293. In this calculation results of the standard lipid panel are first used to estimate LDL-C by the Sampson equation (*Sampson* LDL-C) [23] and then lbLDL-C is calculated with a second equation that depends upon LDL-C and TG.

To further assess lipoprotein subclass profiles along with GlycA, the automated Vantera clinical NMR analyzer (Labcorp, NC, USA) was used. The LipoProfile-4 algorithm was applied for measuring the lipoprotein subclass parameters: VLDL particle size (VLDL-Z) and number (VLDL-P), triglyceride-rich lipoproteins particle (TRL-P) and the following sub-fractions: very small-, small-, medium- and large-TRL-P; LDL particle sizes (LDL-Z) and number (LDL-P), as well as their subfractions: small-, medium-, large-LDL-P; HDL particle size (HDL-Z) and number (HDL-P), as well as their subfractions: small-HDL-P (HDL-P1 ~ 2), medium-HDL-P (HDL-P3 ~ 4), and large-HDL-P (HDL-P5 ~ 7).

### Coronary artery imaging

All subjects underwent CCTA on the same day as the blood draw, utilizing the same CT scanner (320-dectector row Aquilion ONE ViSION, Toshiba, Japan). Radiation exposure was in accordance with the National Institutes of Health Radiation Exposure Committee guidelines. Images were acquired at a slice thickness of 0.5 mm with a slice increment of 0.25 mm. CCTA scan evaluation was done based on the CAD-RADS classification [25]. Severe CAD was defined as total coronary occlusion (CAD-RADS 5) and non-CAD was defined as no significant stenosis or minimum stenosis (CAD-RADS 0 or CAD-RADS 1). All scans were reviewed for quality and for presence of artifacts that would preclude a reliable qualitative and quantitative evaluation. Coronary artery burden adjusted for luminal attenuation was determined across each of the three main coronary arteries by means of a semi-automated software QAngio CT (Medis, Netherlands) [26]. Manual adjustment of inner lumen and outer vessel wall delineations was performed if needed. Total coronary artery burden (TB) and non-calcified coronary artery burden (NCB) indices (mm^2^) were calculated by dividing total vessel plaque volume by total vessel length. Total plaque burden was defined as the sum of calcified plaque burden and non-calcified plaque burden. Non-calcified plaque subcomponents, including fibrous (FB), fibro-fatty (FFB) and necrotic core (NB), were obtained after adaptively correcting for lumen attenuation and depicted based on Hounsfield Units.

CAC was calculated by an experienced cardiologist, using semiautomated software (SmartScore, GE Healthcare). CAC (mean total Agatston score) was measured using electron beam tomography from 40 continuous 3-mm thick computed tomograms (Imatron, CA, USA). A single, experienced radiological technologist performed scoring, blinded to clinical and laboratory data, using customized software (Imatron). Natural log-transformation of CAC scores, (ln[CAC + 1]), was performed to account for the high percentage of CAC scores of 0 in all groups [27].

### Statistical analysis

Data were reported as the mean ± SD for parametric variables or the median (IQR) for non-parametric variables, and as number (%) for categorical variables. Shapiro-Wilk test was considered to assess normality. Multiple groups comparison was done by ANOVA. Spearman correlation was applied for comparison between CCTA plaque characteristics and estimated sdLDL-C. To assess the predictive utility of estimated sdLDL-C and lbLDL-C values on coronary artery burden, a univariable linear regression modeling was applied per quartiles (Q1-Q4) of each estimated lipoprotein plasma concertation with *P* values adjusted based on the Bonferroni method to control for the type I error of multiple comparison.

In the utilized multivariable regression analyses models, adjustment was done for covariates by including traditional cardiovascular risk factors, such as age, sex, current smoking, body mass index (BMI), statin treatment, LDL-C and TGs. Standardized β-coefficient values along with *P*-values were reported for these analyses. *P* values ≤ 0.05 were considered statistically significant.

Parametric and nonparametric (Friedman) repeated measurement ANOVA was used to compare demographic and clinical characteristics of the psoriasis cohort over time for continuous and generalized linear mixed effect model for categorical variables, respectively. To further determine longitudinal prognostic value of estimated sdLDL-C on coronary artery burden, multivariate logistic regression analysis was conducted to model the CCTA plaque progression over 4 years. Receiver operating characteristic (ROC) curve analysis with area under the curve (AUC) was presented to compare the predictive capability of sdLDL-C, LDL-C and ApoB based on a randomized permutation test. The base model was initially adjusted for age and BMI. Wald test was applied to test the significance of sdLDL-C in the logistic regression model. This analysis was conducted in 210 coronary arteries (75 subjects) over 4 years follow-up. Statistical analyses were performed using Stata/IC 12.0 (StataCorp LP, TX, USA) and (R-software version 4.0.5).

## Results

### Baseline characteristics of the study cohort

Psoriasis cohort was represented by predominantly male (64.5%) middle-aged (53.7 ± 10.6 years) and overweight (27.79 ± 3.90) subjects (Table 1). Most of the cohort (77.2%) had no significant or only minimal stenosis (CAD-RADS 0 or CAD-RADS 1) (data not shown), with post-percutaneous coronary intervention (PCI) occurring in only 4.0%. Enrolled patients had moderate skin disease with a mean PASI score of 7.73 (3-9.75) and 26% received some biologic treatment at baseline. The mean LDL-C of the patients was not elevated (104 ± 33.2 mg/dL) and they tended to be more inflamed, as defined by high hsCRP (2.95 mg/L) and GlycA (398.59 µmol/L) values (Table 1).

**Table 1:** Demographic and clinical characteristics of the study cohort

Parameter	N = 200
**Demographics and medical history**	
Age (years)	53.7 ± 10.6
Male sex, n (%)	129 (64.5)
Body mass index (kg/m^2^)	27.79 ± 3.90
Hypertension, n (%)	55 (27.5)
Type 2-diabetes, n (%)	19 (9.5)
Current smoker, n (%)	20 (10.0)
Statin treatment, n (%)	53 (26.5)
Post-PCI, n (%)	8 (4.0)
PASI score	7.73 (3-9.75)
Disease duration (years)	21.3 ± 13.8
Nonbiologic systemic treatment, n (%)	24 (12.0)
Biologic treatment, n (%)	52 (26.0)
**Clinical and laboratory values**	
Total cholesterol (mg/dL)	185.76 ± 41.25
HDL cholesterol (mg/dL)	57.83 ± 19.84
LDL cholesterol (mg/dL)	104.19 ± 33.29
Triglycerides (mg/dL)	99.00 (69.5-133.5)
Remnant cholesterol (mg/dL)	25.31 (14–27)
ApoA-I (mg/dL)	154.87 ± 33.19
ApoB (mg/dL)	91.94 ± 24.61
ApoB / ApoA-I	0.61 ± 0.20
hsCRP (mg/L)	2.95 (0.7-3.0)
GlycA (µmol/L)	398.59 (351–441)
**NMR profile**	
LDL Particle	1417.03 (1093.29-1722.92)
L-LDLP	304.34 (156.43-453.24)
M-LDLP	211.95 (38.44-447.12)
S-LDLP	679.51 (459.99 -1023.57)
HDL Particle	20.75 (18.85–23.25)
L-HDLP	1.92 (1.14–3.13)
M-HDLP	3.91 (2.23–5.07)
S-HDLP	15.08 (12.57–17.76)
TG-Rich LP	115.73 (64.51- 163.73)
Very Large TRLP	0.08 (0.04–0.16)
Large TRLP	1.05 (0.08-3.00)
Medium TRLP	9.72 (3.02–18.19)
Small TRLP	42.52 (20.34–66.85)
Very Small TRLP	40.18 (16.48–83.64)
**Equation-based lipid parameters**	
Estimated lb (buoyant) LDL-C (mg/dL)	74.02 ± 24.47
Estimated sdLDL-C (mg/dL)	32.60 ± 13.00
Estimated LDL-C (mg/dL)	106.62 ± 33.87
**CCTA parameters and CAC score**	
Total plaque burden (x100), mm^2^	1.17 ± 0.46
Non-calcified plaque burden (x100), mm^2^	1.10 ± 0.43
lnCAC score	0 (0-11.75)
Fibrous burden (x100), mm^2^	0.82 ± 0.35
Fibro-fatty burden (x100), mm^2^	0.14 (0.05–0.18)
Necrotic burden (x100), mm^2^	0.028 (0.003–0.017)

Data represented as mean ± SD or median (IQR) for parametric and non-parametric variables respectively and as n (%) for categorical variables. hs(CRP); high-sensitivity C-reactive protein; CAC, Agatston score

### Association of the estimated sdLDL-C with CCTA plaque parameters

Association of the estimated sdLDL-C with coronary plaque characteristics as determined by the CCTA in comparison to other lipoproteins was examined. In unadjusted analysis, sdLDL-C was positively associated with noncalcified plaque burden (NCB) (β = 0.099; *P* = 0.016) and fibro-fatty burden (FFB) (β = 0.117; *P* = 0.005) (Table 2). Although some of the observed significant correlations with sdLDL-C were relatively weak, LDL-C as determined by the Friedewald or Sampson equations was not able to capture any of the investigated associations with CCTA parameters. In contrast, ApoB and NMR-based LDL particle number (LDL-P) were also positively associated with several of the CCTA parameters (Table 2). Finally, we observed a negative association of lbLDL-C with CAC (β=-0.081; *P* = 0.047).

**Table 2:** Univariate Spearman correlation between CCTA plaque characteristics, estimated sdLDL-C and other lipoproteins at the baseline

Variable	Estimated sdLDL-C	Estimated lbLDL-C	LDL-C (*Sampson*)	LDL-C (*Friedewald*)	ApoB	LDL-P
**TB**	0.058 (0.159)	-0.053 (0.201)	-0.004 (0.931)	-0.019 (0.650)	0.032 (0.449)	0.060 (0.187)
**NCB**	0.099 (**0.016**)	-0.006 (0.877)	0.044 (0.291)	0.029 (0.492)	0.066 (0.118)	0.112 (**0.013**)
**CAC**	-0.013 (0.744)	-0.081 (**0.047**)	-0.069 (0.093)	-0.086 (0.039)	-0.055 (0.188)	-0.107 (**0.017**)
**Plaque morphology index**						
**Fibrous burden (mm** ^**2**^ **)**	0.051 (0.221)	-0.052 (0.218)	-0.017 (0.681)	-0.028 (0.513)	-0.028 (0.508)	0.022 (0.631)
**Fibro-fatty burden (mm** ^**2**^ **)**	0.117 (**0.005**)	0.024 (0.572)	0.067 (0.108)	0.061 (0.146)	0.120 (**0.005**)	0.168 (**0.0002**)
**Necrotic burden (mm** ^**2**^ **)**	0.063 (0.169)	0.069 (0.135)	0.072 (0.114)	0.078 (0.093)	0.110 (**0.017**)	0.115 (**0.019**)

Results from Spearman correlation were reported as *r* coefficient (*P* values). *P* ≤ 0.05 considered significant. TB, total burden; NCB, non-calcified burden; CAC, Agatston score

Next, how estimated sdLDL-C might affect the detected associations with coronary plaque characteristics based on plasma levels quartiles analysis was tested (Supplemental Table 1). Univariable linear regression analysis revealed a strong positive association with fibrous burden (FB) (β = 0.22; *P* = 0.04) in the highest sdLDL-C quartile (Q4) (Supplemental Table 1 A). In contrast, a negative association between the highest estimated sdLDL-C quartiles (Q3) and CAC was observed. Quartiles-based analyses of the CCTA plaque parameters showed insignificant trends for NCB, FFB and NB rise in the higher psoriasis quartiles (Q3-Q4) (Supplemental Table 1B). Estimated large lbLDL-C was also positively associated with total coronary plaque burden (TB) and NCB in Q2 (Supplemental Table 2). The observed associations with estimated sdLDL-C remained evident for most of the CCTA plaque characteristics, except NB, after adjusting for LDL-C (Table 3). However, in fully adjusted models (age, sex, smoking, BMI, statin treatment, LDL-C and TGs) the investigated association only remained statistically significant for only TB (β = 0.54; *P* = 0.005) and NCB (β = 0.37; *P* = 0.050) (Table 3 and Supplemental Table 3).

**Table 3:** Multivariable adjusted association between CCTA plaque characteristics and estimated sdLDL-C at the baseline

	TB	NCB	CAC	FB	FFB	NB
**Model 1**	0.26 (**< 0.0001**)	0.27 (**< 0.0001**)	0.15 (**0.037**)	0.22 (**0.002**)	0.29 (**< 0.0001**)	0.09 (0.28)
**Model 2**	0.54 (**0.005**)	0.37 (**0.050**)	-0.01 (0.98)	0.28 (0.19)	0.20 (0.32)	0.19 (0.48)

Model 1: Adjusted for LDL-C.

Model 2: Adjusted for age, sex, smoking, BMI, statin treatment, LDL-C, TGs.

Results from multivariable linear regression models were reported as standardized β coefficient (*P* values). *P* ≤ 0.05 considered significant. CAC was log-transformed. TB, total burden; NCB, non-calcified burden; CAC, Agatston score. FB, fibrous burden; FFB, fibro-fatty burden; NB, necrotic burden

Finally, characterization of which estimated sdLDL particles may contribute the most to the observed associations was assessed in comparison to other lipoproteins based on the NMR profile (Supplemental Table 4). By this analysis, small LDLP (S-LDLP) and S-HDLP along with large and medium TG-rich lipoproteins (TRLPs) had the most significant positive correlations.

### Predictive value of estimated sdLDL-C over time in psoriasis

**Fig. 1:**
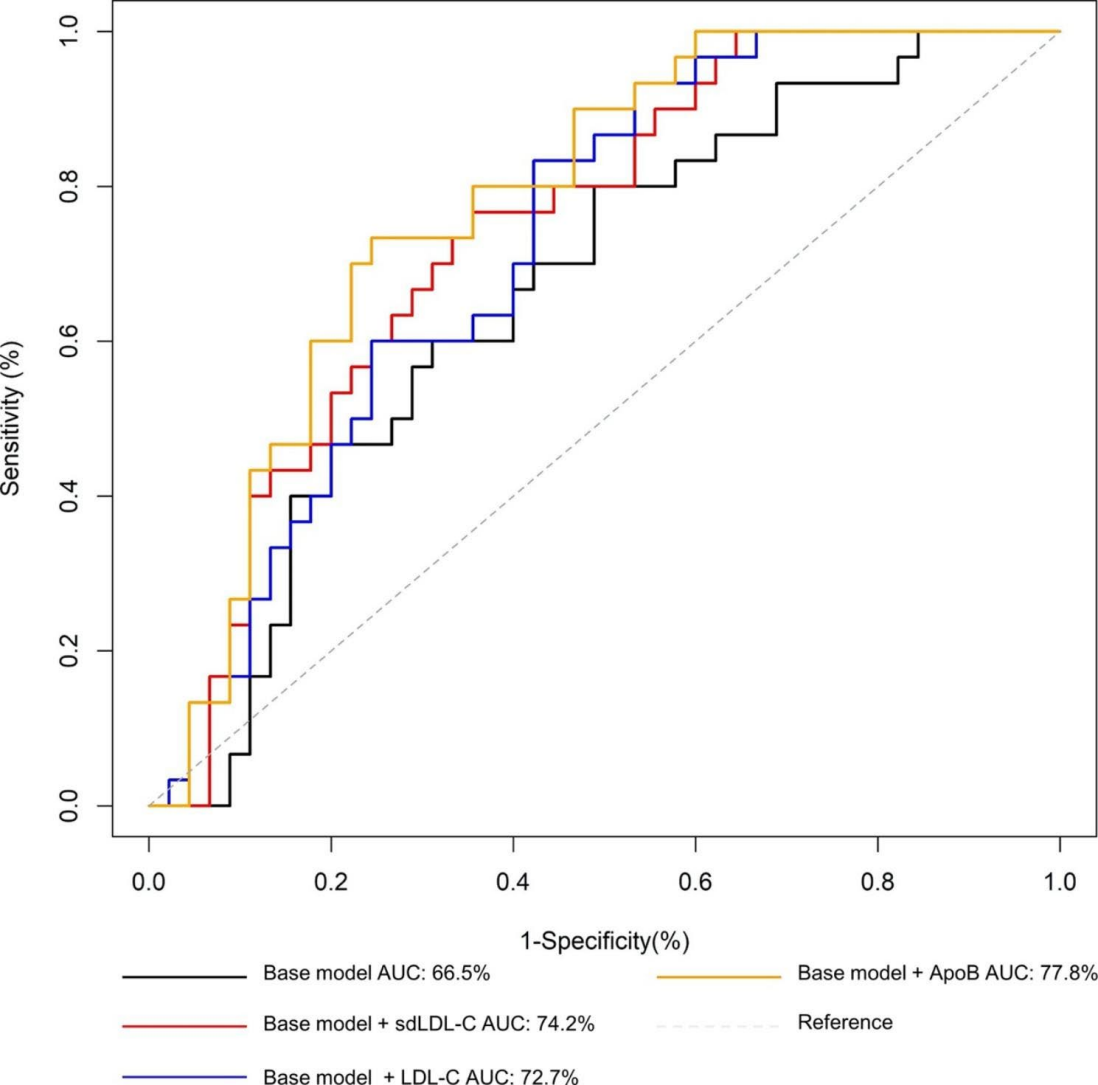
Results comparing area under curve (AUC) of ROC based on logistic regression modelling 4-year follow-up data. Base model included age and BMI. *P* ≤ 0.05 considered significant

To further investigate psoriasis-specific effects on sdLDL-C and related coronary plaque progression, a small sub-cohort of psoriasis patients (N = 75) followed over 4 years was examined. During this time, the most robust changes were reduction of PASI score and GlycA, which might be explained by effective biologic treatment (Supplemental Table 5). These changes were also accompanied by decreases in ApoA-I, along with significantly lower S-LDLP and TRLPs. Additionally, CCTA markers of plaque instability, FFB (23%) and NC (14%), were moderately higher when comparing baseline to the 4-year follow-up time point (Table 4). To further explore a potential contribution of estimated sdLDL-C in assessing plaque vulnerability parameters, receiver operating characteristic (ROC) analysis was performed (N = 210 arteries). Based on ROC plot analysis of the small sub-cohort who had a 4-year follow-up CCTA study, estimated sdLDL-C resulted in a slightly better AUC value for the NB among other investigated CCTA high-risk coronary plaque features, when added to the base model adjusted for age and BMI (AUC of sdLDL-C: 74.2% vs. AUC of LDL-C: 72.7%; *P* = 0.60) (Fig. 1). Although the observed AUC differences were not statistically significant, by regression modelling estimated sdLDL-C did significantly predict NB progression over the follow-up period (*P* = 0.015), whereas LDL-C did not. Of note, ApoB had a higher AUC value than the other tests.

**Table 4:** Changes in the CCTA plaque characteristics, estimated sdLDL-C and LDL-C during 1- and 4-year follow-up in study cohort

Variable	Baseline N = 210 arteries	1 year N = 210 arteries	4 years N = 210 arteries	*P*
Total plaque burden (x100), mm^2^	1.17 ± 0.51	1.20 ± 0.57	1.11 ± 0.40	0.605
Non-calcified plaque burden (x100), mm^2^	1.10 ± 0.50	1.14 ± 0.58	1.05 ± 0.38	0.727
Fibrous burden (x100), mm^2^	0.94 ± 0.43	0.89 ± 0.42	0.57 ± 0.25	**< 0.001**
Fibro-fatty burden (x100), mm^2^	0.13 ± 0.12	0.17 ± 0.17	0.16 ± 0.16	0.066
Necrotic burden (x100), mm^2^	0.07 ± 0.27	0.03 ± 0.08	0.08 ± 0.15	**0.034**
Estimated sdLDL-C (mg/dL)	33.14 ± 11.58	31.72 ± 12.10	32.98 ± 12.74	0.116
LDL-C (mg/dL)	104.97 ± 35.96	101.41 ± 37.67	107.56 ± 36.05	0.188

Data represented as mean ± SD. *P* values were derived from repeated measurement ANOVA. *P* ≤ 0.05 considered significant

## Discussion

Although the standard lipid panel is valuable for calculating LDL-C and is also used for assessing 10-year ASCVD risk, this approach has limitations [8]. In particular, estimating LDL-C by the Friedewald equation, which is still the most commonly used equation by the College of American Pathology proficiency test reports, has been reported to be inaccurate especially in patients with low LDL-C [28] or high TGs blood levels [29]. Furthermore, chronic inflammatory conditions, such as psoriasis, are known to affect lipoprotein metabolism, enhance atherosclerotic plaque progression and contribute to high CVD risk [30]. A psoriasis-specific lipid phenotype has been characterized by relatively low LDL-C before the onset of symptoms [6] and is also accompanied by increases in TGs during disease progression [7]. Although the exact biological mechanisms behind this phenomenon has never been fully explored, some reports suggest a possible contribution of type 2 diabetes [31] and metabolic syndrome [32], because they are common co-morbid conditions in PSO.

Impaired TGs metabolism is one of the key pathophysiological factors shared by all the conditions in which LDL-C has been found to have a weaker association with ASCVD. In the setting of hypertriglyceridemia, excessive TGs are exchanged between TGs-rich LDL, large lbLDL and TRLPs through cholesteryl ester transfer protein (CETP) exchange [33], resulting in triglyceride-rich lipoprotein cholesterol (remnant cholesterol) increase. Subsequently, TGs contained in LDL undergo further lipolysis by lipoprotein and hepatic lipases culminating in increased levels of sdLDL-C [34]. In contrast, LDL-C is typically lowered by lipolysis because the levels of lbLDL decrease, and it is this large LDL subfraction that has the greatest carrying capacity for cholesterol. This inverse relationship of LDL-C with hypertriglyceridemia limits the value of LDL-C as univariate test for initial ASCVD risk stratification.

The prognostic value of sdLDL-C and remnant cholesterol have been assessed in recent large clinical studies, indicating it has potential value for estimating CVD risk [21] and in characterizing inflammation [19]. Some of the biophysical features of sdLDL, such as higher susceptibility to oxidation in vitro [16] and increase uptake by macrophages [13], could explain its role in atherogenesis and plaque formation. Thus, sdLDL-C might be elevated in chronic inflammatory conditions, such as psoriasis, and could be a promising biomarker for the development of CAD in psoriasis. Indeed, a significant association between higher levels of estimated sdLDL-C and coronary plaque burden, as assessed by NCB values, was revealed in the current study. Moreover, vulnerable plaque morphological characteristics, such as fibrous burden and necrotic burden, were also positively associated with estimated sdLDL-C over time. These results support previously reported data suggesting that patients with psoriasis develop unstable coronary plaques, characterized by thin fibrous cap and large lipid-rich necrotic core, which can possibly be favorably modified under effective biologic treatment [35].

Besides the biological properties of sdLDL already mentioned above, its smaller size permits greater access to the intimal space with subsequent subintimal retention and then also uptake by cells. Moreover, the longer clearance time from the circulation when compared to the lbLDL particles might also explain the compositional changes of sdLDL particles, such as increased content of oxidized lipids and increased accumulation of TGs through the CETP-mediated cholesteryl ester exchange.

The NMR-based lipid profile in the current study revealed a significant positive association of estimated sdLDL-C with S-LDLP and TRLPs, particularly large and medium TRLPs, in patients with psoriasis. These observations might be in part explained by the apolipoprotein content of triglyceride-related lipoproteins, such as ApoE or ApoC-III, and their ability to modulate lipoprotein lipase activity and TRL metabolism. For example, ApoE is known to affect the metabolism of ApoB-containing lipoproteins by modulating the interaction of lipoproteins with LDL and VLDL receptors and thus affecting hepatic clearance [36]. This interaction is also under the influence of proinflammatory ApoC-III, which is found on TRLPs and on HDL. A rise in plasma TGs from high ApoC-III is also known to inhibit the clearance of TRLPs from the plasma and to accelerate the catabolism of HDL [37]. As a result, combination of ApoC-III with ApoE in HDL, may negatively affect the antiatherogenic characteristics of HDL [38] and coronary plaque phenotype [39]. Indeed, HDL-C concentration itself does not solely determine CVD risk, as HDL particles cholesterol content and size also modifies risk [40]. In line with these discoveries, S-HDLP was strongly associated with estimated sdLDL-C, suggesting that small HDL particles along with TRLPs might play a role in the observed coronary plaque changes. The discussed alterations in TG metabolism and potential clinical effectiveness of newly developed drugs, such as ApoC-III inhibitors, deserve further exploration in psoriasis [41].

### Study strengths

To our knowledge, this is the first prospective study investigating estimated sdLDL-C in psoriasis. The potential prognostic value of estimated sdLDL-C over LDL-C in assessing CAD as determined by coronary plaque burden over time was demonstrated, as well as important insights were provided on TG metabolism and dyslipidemia in psoriasis.

### Study limitations

It is important to note that the current study was relatively small, particularly the sub-cohort followed prospectively. Furthermore, the strength of the associations of estimated sdLDL-C with various CCTA characteristics decreased after multivariate adjustments. Nevertheless, in most current guidelines LDL-C is used in a univariate fashion to perform initial ASCVD risk stratification. Larger studies, however, will be needed that compare estimated sdLDL-C to LDL-C with clinical ASCVD events to fully understand the possible clinical utility of sdLDL-C. It would also be interesting to determine if the association of estimated sdLDL-C with ASCVD is stronger in psoriasis and other inflammatory diseases compared to a control group without inflammation. Another limitation to the current study is that sdLDL-C was not directly measured but instead estimated. Thus, it will be important in the future to compare estimated and directly measured sdLDL-C, but estimated sdLDL-C was a better predictor of clinical ASCVD events than measured sdLDL-C in one previous study [23].

## Conclusion

In patients with psoriasis, a newly developed equation-based sdLDL-C estimate derived from the standard lipid panel had a stronger association with several CCTA parameters than LDL-C. Because sdLDL-C and LDL-C are calculated using the same standard lipid panel tests, these findings can be readily translated. More studies, however, in larger populations with primary endpoints based on clinical events will be needed to establish if estimated sdLDL-C can be used more broadly in the clinical care of patients.

## Electronic Supplementary Material

Below is the link to the electronic supplementary material.

Supplementary Material 1

## Data Availability

De-identified patient data can be available upon reasonable request to the corresponding author.

## Abbreviations

ApoE: Apolipoprotein E
ApoC-III: Apolipoprotein C-III
ASCVD: Atherosclerotic cardiovascular disease
CAC: Coronary artery calcium
CAD: Coronary artery disease
CAD-RADS: Coronary artery disease-reporting and data system
CCTA: Coronary computed tomography angiography
FB: Fibrous burden
FFB: Fibro-fatty burden
NB: Necrotic burden
NCB: Noncalcified plaque burden
PCI: Percutaneous coronary intervention
PSO: Psoriasis
sdLDL-C: Small dense low-density lipoprotein-cholesterol
sLDL: Small low-density lipoprotein
S-LDLP: Small low-density lipoprotein particle
TRLP: Triglyceride-rich lipoprotein
