# Supplementary material for: Estimated sdLDL-C for predicting high-risk coronary plaque features in psoriasis: a prospective observational study
Source: Lipids Health Dis. 2023 Apr 27;22:55. doi: 10.1186/s12944-023-01819-x (PMC10134516; doi:10.1186/s12944-023-01819-x)
Supplement: Supplementary file 1 — Supplementary Material 1 [file 12944_2023_1819_MOESM1_ESM.docx]

**Supplemental Information**


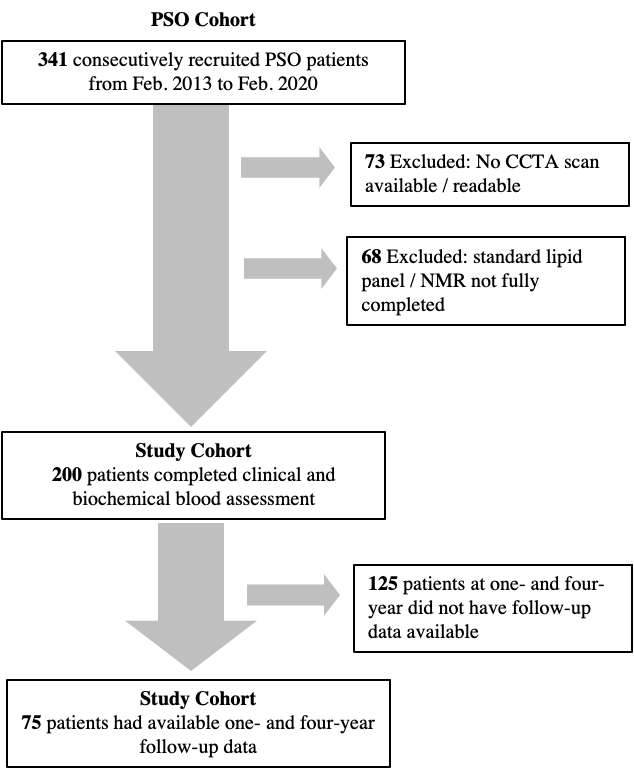


**Supplemental Figure 1.** Recruitment and follow-up scheme of study participants. CCTA, coronary computed tomography angiography; PSO, psoriasis.

**Supplemental Table 1.** Association between CCTA plaque characteristics and estimated sdLDL-C in study cohort according to quartiles at the baseline

**(A)**

| **Variable** | **sdLDL-C** | | | |
| --- | --- | --- | --- | --- |
|  | **Q1**  **46, 18.54** ± **2.69** | **Q2**  **48, 26.12** ± **2.05** | **Q3**  **50, 33.59** ± **2.25** | **Q4**  **56, 48.83** ± **11.24** |
| **CCTA** |  |  |  |  |
| **TB** | -0.12 (0.76) | -0.07 (>0.99) | -0.09 (>0.99) | 0.15 (0.24) |
| **NCB** | -0.13 (0.60) | -0.07 (>0.99) | -0.07 (>0.99) | 0.17 (0.12) |
| **CAC** | 0.18 (0.12) | -0.20 (0.08) | -0.30 (**0.0004**) | -0.18 (0.40) |
| **Plaque morphology index** |  |  |  |  |
| **Fibrous burden (mm^2^)** | -0.07 (>0.99) | -0.09 (>0.99) | -0.18 (0.12) | 0.22 (**0.04**) |
| **Fibro-fatty burden (mm^2^)** | -0.06 (>0.99) | 0.002 (>0.99) | -0.03 (>0.99) | 0.05 (>0.99) |
| **Necrotic burden (mm^2^)** | -0.06 (>0.99) | 0.15 (0.40) | -0.07 (>0.99) | 0.04 (>0.99) |

Results from univariable linear regression models were reported as standardized β coefficient (*P* values). *P* values were adjusted based on the Bonferroni correction. *P*≤0.05 considered significant. TB, total burden; NCB, non-calcified burden; CAC, Agatston score.

**(B)**

| **Variable** | **sdLDL-C** | | | | |
| --- | --- | --- | --- | --- | --- |
|  | **Q1**  **46, 18.54** ± **2.69** | **Q2**  **48, 26.12** ± **2.05** | **Q3**  **50, 33.59** ± **2.25** | **Q4**  **56, 48.83** ± **11.24** | ***P*** |
| **CCTA** |  |  |  |  |  |
| **TB** | 1.20 ± 0.51 | 1.09 ± 0.46 | 1.19 ± 0.39 | 1.20 ± 0.47 | 0.30 |
| **NCB** | 1.07 ± 0.40 | 1.04 ± 0.46 | 1.14 ± 0.41 | 1.15 ± 0.44 | 0.31 |
| **CAC** | 0 (0 - 2) | 0 (0 - 22) | 0 (0 - 25) | 0 (0 - 8) | 0.86 |
| **Plaque morphology index** |  |  |  |  |  |
| **Fibrous burden (mm^2^)** | 0.0088 ± 0.0036 | 0.0085 ± 0.0038 | 0.0087 ± 0.0029 | 0.0087 ± 0.0025 | 0.49 |
| **Fibro-fatty burden (mm^2^)** | 0.0013 ± 0.0009 | 0.0014 ± 0.0010 | 0.0016 ± 0.0012 | 0.0019 ± 0.0016 | 0.59 |
| **Necrotic burden (mm^2^)** | 0.0005 ± 0.0008 | 0.0003 ± 0.0004 | 0.0010 ± 0.0023 | 0.0006 ± 0.0012 | 0.65 |

Data represented as mean ± SD. *P* values were derived from ANOVA. *P*≤0.05 considered significant. TB, total burden; NCB, non-calcified burden; CAC, Agatston score.

**Supplemental Table 2.** Association between CCTA plaque characteristics and estimated lbLDL-C in study cohort according to quartiles at the baseline

| **Variable** | **lbLDL-C** | | | |
| --- | --- | --- | --- | --- |
|  | **Q1**  **37, 39.33 ± 9.75** | **Q2**  **49, 62.13 ± 4.82** | **Q3**  **56, 77.36 ± 4.90** | **Q4**  **58, 102.97 ± 14.84** |
| **CCTA** |  |  |  |  |
| **TB** | -0.32 (**0.004**) | 0.26 (**0.008**) | 0.19 (0.08) | -0.03 (0.70) |
| **NCB** | -0.32 (**0.004**) | 0.28 (**0.004**) | 0.19 (0.08) | -0.04 (0.63) |
| **CAC** | 0.11 (>0.99) | 0.07 (>0.99) | -0.05 (>0.99) | -0.15 (0.20) |
| **Plaque morphology index** |  |  |  |  |
| **Fibrous burden (mm^2^)** | -0.25 (**0.04**) | 0.21 (**0.04**) | 0.10 (0.76) | -0.01 (>0.99) |
| **Fibro-fatty burden (mm^2^)** | -0.22 (0.12) | 0.18 (0.12) | 0.08 (>0.99) | -0.01 (>0.99) |
| **Necrotic burden (mm^2^)** | -0.16 (0.13) | 0.21 (0.08) | 0.07 (0.42) | -0.02 (>0.99) |

Results from univariable linear regression models were reported as standardized β coefficient (*P* values). *P* values were adjusted based on the Bonferroni correction. *P*≤0.05 considered significant. TB, total burden; NCB, non-calcified burden; CAC, Agatston score.

**Supplemental Table 3.** Association between plaque characteristics and estimated sdLDL-C in study cohort at the baseline

| **Model** | **sdLDL-C** |
| --- | --- |
|  | **Total plaque burden (x100), mm^2^** |
| Adjusted for age | 0.06; 0.13 |
| Adjusted for sex | 0.04; 0.26 |
| Adjusted for current smoking | 0.05; 0.23 |
| Adjusted for BMI | -0.06; 0.12 |
| Adjusted for statin treatment | 0.06; 0.17 |
| Adjusted for LDL-C | 0.26; **<0.0001** |
| Adjusted for TGs | -0.02; 0.73 |
| Adjusted for age, sex, smoking, BMI, statin treatment, LDL-C, TGs | 0.54; **0.005** |
|  | **Non-calcified plaque burden (x100), mm^2^** |
| Adjusted for age | 0.10; **0.02** |
| Adjusted for sex | 0.09; 0.02 |
| Adjusted for current smoking | 0.10; **0.02** |
| Adjusted for BMI | -0.03; 0.50 |
| Adjusted for statin treatment | 0.10; **0.02** |
| Adjusted for LDL-C | 0.27; **<0.0001** |
| Adjusted for TGs | 0.03; 0.65 |
| Adjusted for age, sex, smoking, BMI, statin treatment, LDL-C, TGs | 0.37; **0.050** |
|  | **CAC score** |
| Adjusted for age | 0.02; 0.57 |
| Adjusted for sex | -0.06; 0.15 |
| Adjusted for current smoking | -0.05; 0.19 |
| Adjusted for BMI | -0.06; 0.20 |
| Adjusted for statin treatment | -0.01; 0.72 |
| Adjusted for LDL-C | 0.15; **0.04** |
| Adjusted for TGs | -0.17; **0.01** |
| Adjusted for age, sex, smoking, BMI, statin treatment, LDL-C, TGs | -0.01; 0.98 |
|  | **Fibrous burden (mm^2^)** |
| Adjusted for age | 0.04; 0.32 |
| Adjusted for sex | 0.03; 0.44 |
| Adjusted for current smoking | 0.04; 0.39 |
| Adjusted for BMI | -0.06; 0.14 |
| Adjusted for statin treatment | 0.04; 0.38 |
| Adjusted for LDL-C | 0.22; **0.002** |
| Adjusted for TGs | -0.05; 0.46 |
| Adjusted for age, sex, smoking, BMI, statin treatment, LDL-C, TGs | 0.28; 0.19 |
|  | **Fibro-fatty burden (mm^2^)** |
| Adjusted for age | 0.14; **0.001** |
| Adjusted for sex | 0.15; **<0.0001** |
| Adjusted for current smoking | 0.15; **<0.0001** |
| Adjusted for BMI | 0.04; 0.33 |
| Adjusted for statin treatment | 0.16; **<0.0001** |
| Adjusted for LDL-C | 0.29; **<0.0001** |
| Adjusted for TGs | 0.14; **0.03** |
| Adjusted for age, sex, smoking, BMI, statin treatment, LDL-C, TGs | 0.20; 0.32 |
|  | **Necrotic burden (mm^2^)** |
| Adjusted for age | 0.04; 0.35 |
| Adjusted for sex | 0.04; 0.34 |
| Adjusted for current smoking | 0.05; 0.33 |
| Adjusted for BMI | 0.01; 0.89 |
| Adjusted for statin treatment | 0.04; 0.41 |
| Adjusted for LDL-C | 0.09; 0.28 |
| Adjusted for TGs | 0.05; 0.48 |
| Adjusted for age, sex, smoking, BMI, statin treatment, LDL-C, TGs | 0.19; 0.48 |

Results from multivariable linear regression models were reported as standardized β coefficient (*P* values). *P*≤0.05 considered significant. CAC was log-transformed. CAC, Agatston score.

**Supplemental Table 4.** Univariate Spearman correlation between estimated sdLDL-C, ApoB and LDL particle concentrations at the baseline

| **Variable** | **sdLDL-C** | **ApoB** | **LDL-P** |
| --- | --- | --- | --- |
| **LDL Particle** | 0.644; **<0.001** | 0.758; **<0.001** | - |
| L-LDLP | 0.191; **0.014** | 0.291; **<0.001** | 0.324; **<0.001** |
| M-LDLP | 0.006; 0.936 | 0.139; 0.079 | 0.308; **<0.001** |
| S-LDLP | 0.408; **<0.001** | 0.380; **<0.001** | 0.549; **<0.001** |
| **HDL Particle** | 0.140; 0.072 | 0.192; **0.015** | 0.275; **<0.001** |
| L-HDLP | -0.334; **<0.001** | -0.203; **0.010** | -0.149; 0.056 |
| M-HDLP | -0.020; 0.795 | 0.012; 0.879 | 0.020; 0.799 |
| S-HDLP | 0.277; **<0.001** | 0.280; **<0.001** | 0.329; **<0.001** |
| **TG-Rich LP** | 0.681; **<0.001** | 0.495; **<0.001** | 0.419; **<0.001** |
| Very Large TRLP | -0.292; **<0.001** | -0.266; **0.001** | -0.242; **0.002** |
| Large TRLP | 0.396; **<0.001** | 0.217; **0.006** | 0.181; **0.020** |
| Medium TRLP | 0.419; **<0.001** | 0.198; **0.012** | 0.163; **0.036** |
| Small TRLP | 0.333; **<0.001** | 0.228; **0.004** | 0.078; 0.319 |
| Very Small TRLP | 0.379; **<0.001** | 0.260; **0.001** | 0.280; **<0.001** |

Results from Spearman correlation were reported as *r* coefficient. *P*≤0.05 considered significant.

**Supplemental Table 5.** Demographic and clinical characteristics of the study cohort over time

| **Parameter** |  |  |  |  |
| --- | --- | --- | --- | --- |
| **Demographics and medical history** | **Baseline, n=75** | **1 year, n=75** | **4 years, n=75** | ***P*** |
| Age (years) | 54.0 ± 10.0 | 55.3 ± 9.8 | 58.3 ± 10.0 | **<0.001** |
| Male sex, n (%) | 50 (67) | 50 (67) | 50 (67) | >0.999 |
| Body mass index (kg/m^2^) | 27.67 ± 3.55 | 27.68 ± 3.66 | 27.87 ± 3.86 | 0.028 |
| Hypertension, n (%) | 15 (20) | 16 (22) | 17 (23) | 0.456 |
| Type 2-diabetes, n (%) | 4 (5) | 4 (5) | 8 (11) | **0.014** |
| Current smoker, n (%) | 7 (9) | 3 (4) | 3 (5) | 0.246 |
| Statin treatment, n (%) | 21 (28) | 18 (25) | 21 (31) | 0.200 |
| PASI score | 4.3 (2.8 - 8.3) | 3.1 (1.5 - 4.8) | 2.0 (0.6 - 4.4) | **<0.001** |
| Nonbiologic systemic treatment, n (%) | 9 (12) | 8 (11) | 9 (12) | 0.738 |
| Biologic treatment, n (%) | 27 (36) | 41 (55) | 39 (53) | **0.002** |
| **Clinical and laboratory values** |  |  |  |  |
| Total cholesterol (mg/dL) | 183.73 ± 39.89 | 183.59 ± 43.01 | 181.22 ± 41.77 | 0.872 |
| HDL cholesterol (mg/dL) | 55.17 ± 15.52 | 55.48 ± 16.23 | 53.74 ± 13.76 | 0.729 |
| LDL cholesterol (mg/dL) | 104.97 ± 35.96 | 101.41 ± 37.67 | 107.56 ± 36.05 | 0.188 |
| Triglycerides (mg/dL) | 106 (76 - 133) | 107 (75 - 152) | 110 (81 - 159) | 0.509 |
| ApoA-I (mg/dL) | 154.79 ± 30.59 | 155.30 ± 30.76 | 149.38 ± 25.89 | **0.009** |
| ApoB (mg/dL) | 90.35 ± 19.98 | 89.44 ± 23.37 | 94.44 ± 24.85 | **0.005** |
| ApoB / ApoA-I | 0.60 ± 0.17 | 0.60 ± 0.20 | 0.65 ± 0.22 | **<0.001** |
| hsCRP (mg/L) | 2.33 ± 3.14 | 2.08 ± 2.23 | 2.32 ± 3.89 | 0.600 |
| GlycA | 403.09 ± 54.32 | 387.64 ± 56.21 | 379.04 ± 61.54 | **0.017** |
| **NMR profile** |  |  |  |  |
| LDL Particle | 1402.80 (1125.46 - 1673.72) | 1378.15 (1072.20 - 1665.10) | 1084.59 (90.38 - 1495.02) | 0.053 |
| L-LDLP | 308.43 (179.52 - 426.50) | 237.69 (119.95 - 386.24) | 87.46 (53.82 - 348.12) | 0.206 |
| M-LDLP | 169.51 (42.47 - 328.34) | 195.68 (21.76 - 462.54) | 175.67 (130.59 - 424.24) | 0.587 |
| S-LDLP | 683.08 (539.67 - 1077.07) | 776.11 (573.91 - 1053.99) | 355.59 (82.13 - 631.64) | **<0.001** |
| HDL Particle | 20.77 (19.03 - 23.91) | 21.77 (19.66 - 23.95) | 22.72 (20.77 - 86.73) | 0.131 |
| L-HDLP | 1.83 (1.12 - 2.88) | 1.92 (1.33 - 3.50) | 0.73 (0.17 - 2.42) | **<0.001** |
| M-HDLP | 3.70 (1.93 - 5.10) | 3.51 (1.88 - 5.48) | 3.85 (0.80 - 6.36) | 0.508 |
| S-HDLP | 15.26 (12.57 - 17.80) | 15.59 (13.11 - 18.09) | 13.66 (10.10 - 16.42) | 0.187 |
| TG-Rich LP | 115.66 (67.91 - 165.68) | 107.66 (77.69 - 153.33) | 52.04 (36.20 - 122.43) | **<0.001** |
| Very Large TRLP | 0.07 (0.04 - 0.13) | 0.12 (0.04 - 0.25) | 0.17 (0.06 - 132.14) | 0.079 |
| Large TRLP | 1.01 (0.11 - 2.91) | 1.92 (0.63 - 5.85) | 6.56 (0.48 - 96.03) | 0.067 |
| Medium TRLP | 10.78 (4.20 - 18.67) | 11.59 (5.71 - 23.07) | 49.14 (7.91 - 159.33) | **0.003** |
| Small TRLP | 40.55 (20.26 - 74.82) | 41.09 (16.44 - 65.90) | 57.25 (24.85 - 74.89) | 0.208 |
| Very Small TRLP | 39.32 (14.71 - 89.41) | 34.32 (13.04 - 78.67) | 25.37 (7.95 - 47.05) | **0.014** |
| **Equation based lipid parameters** |  |  |  |  |
| Estimated (buoyant) lbLDL-C (mg/dL) | 73.57 (59.13 - 86.89) | 66.35 (52.30 - 81.33) | 64.11 (53.03 - 79.11) | 0.211 |
| Estimated sdLDL-C (mg/dL) | 33.14 ± 11.58 | 31.72 ± 12.10 | 32.98 ± 12.74 | 0.116 |
| **CCTA parameters and CAC score** |  |  |  |  |
| Total plaque burden (x100), mm^2^ | 1.17 ± 0.51 | 1.20 ± 0.57 | 1.11 ± 0.40 | 0.605 |
| Non-calcified plaque burden (x100), mm^2^ | 1.10 ± 0.50 | 1.14 ± 0.58 | 1.05 ± 0.38 | 0.727 |
| Fibrous plaque burden (x100), mm^2^ | 0.94 ± 0.43 | 0.89 ± 0.42 | 0.57 ± 0.25 | **<0.001** |
| Fibro-fatty burden (x100), mm^2^ | 0.13 ± 0.12 | 0.17 ± 0.17 | 0.16 ± 0.16 | 0.066 |
| Necrotic burden (x100), mm^2^ | 0.07 ± 0.27 | 0.03 ± 0.08 | 0.08 ± 0.15 | **0.034** |

Data represented as mean ± SD or median (IQR) for parametric and non-parametric variables respectively and as n (%) for categorical variables. *P* values were derived from repeated measurement ANOVA or nonparametric Friedman test for continuous variables and generalized linear mixed effect model for categorical variables. *P*≤0.05 considered significant. hs(CRP); high-sensitivity C-reactive protein.
